# Regulation of cilium length and intraflagellar transport by the MAP kinases MAK, MRK, and MOK

**DOI:** 10.1186/2046-2530-1-S1-O12

**Published:** 2012-11-16

**Authors:** G Jansen, JR Broekhuis

**Affiliations:** 1 Dept of Cell Biology, Erasmus MC, Roterdam, the Netherlands

## 

Cilia are assembled and maintained by intraflagellar transport (IFT), which moves cargo bi-directionally along the axoneme of the cilium. Regulation of IFT allows modulation of cilia length and influences signaling pathways that initiate in the cilium. We characterized *C. elegans*’ DYF-5, a MAP kinase that regulates cilia length and IFT. DYF-5 has three mammalian homologues: MAK (male germ cell-associated kinase), MRK (MAK-related kinase) and MOK (MAPK/MAK/MRK overlapping kinase). Here we analyzed the functions of MAK, MRK and MOK. GFP fusions of MAK, MRK and MOK showed that all three proteins localize along the length of the cilia of IMCD3 and RPE cells, and accumulate at the distal tip. Knockdown of MRK or MOK resulted in longer cilia, while overexpression of MAK or MRK resulted in short and stumpy cilia. Interestingly, overexpression of MOK did not affect cilia length. These effects on cilia length are in line with our data on DYF-5 function in *C. elegans* (Burghoorn et al., 2007) and published data on MAK (Omori et al., 2010). To visualize IFT and measure its speed we have generated IMCD3 cell lines that stably express GFP-fusion constructs of different components of the IFT complex: Kinesin-II, Kif17, complex A, complex B, and the BBSome. All these GFP fusion proteins showed an average anterograde speed of ~0.4 μm/s and a similar average retrograde speed. Knock down of MOK using shRNAs resulted in increased anterograde speeds of all constructs to ~0.6 μm/s, while knock down of MRK had no effect. Retrograde speeds were not affected.

